# The relationship between early literacy skills and speech-sound production in students with intellectual disability and communication difficulties: a cross-sectional study

**DOI:** 10.1080/20473869.2023.2212958

**Published:** 2023-05-22

**Authors:** Jenny Samuelsson, Jakob Åsberg Johnels, Gunilla Thunberg, Lisa Palmqvist, Mikael Heimann, Monica Reichenberg, Emil Holmer

**Affiliations:** 1 Speech and Language Pathology Unit, Department of Health and Rehabilitation, Institute of Neuroscience and Physiology, Sahlgrenska Academy, University of Gothenburg, Gothenburg, Sweden; 2 Dart - Centre for AAC and Assistive Technology, Sahlgrenska University Hospital, Gothenburg, Sweden; 3 Region Västra Götaland, Habilitation & Health, Habilitation Children and youth, Gothenburg, Sweden; 4 Gillberg Neuropsychiatry Centre, Institute of Neuroscience and Physiology, Sahlgrenska Academy, University of Gothenburg, Gothenburg, Sweden; 5 Child Neuropsychiatric Clinic, Sahlgrenska University Hospital, Gothenburg, Sweden; 6 Disability Research Division, Department of Behavioural Sciences and Learning, Linköping University, Linköping, Sweden; 7 Department of Education and Special Education, University of Gothenburg, Sweden; 8 Division of Psychology, Department of Behavioural Sciences and Learning, Linköping University, Linköping, Sweden

**Keywords:** Intellectual disability, communication difficulties, speech-sound production, early literacy skills, phonological awareness, letter-sound knowledge

## Abstract

Earlier research and reports from educational practice seem to suggest that teaching early literacy skills may facilitate speech-sound production in students with intellectual disabilities, but further research is needed to confirm a potential connection. This study investigated (1) the relationship between speech-sound production, phonological awareness, and letter-sound knowledge in students with intellectual disabilities and communication difficulties, and (2) to what degree phonological awareness and letter-sound knowledge explain the variance in speech-sound production over and above IQ and chronological age. A group of 116 students, aged 7–21, enrolled in Swedish compulsory schools for students with intellectual disabilities participated in this study. All had limited reading skills. The test results for phonological awareness, letter-sound knowledge, and speech-sound production had a wide range. The results showed that early literacy skills were moderately and significantly correlated with speech-sound production. After controlling for IQ and age in a regression model, the addition of phonological awareness and letter-sound knowledge explained 29% of the variance in speech-sound production. The results suggest that phonological awareness and letter-sound knowledge is associated with speech-sound production and that these associations are not explained by age or IQ. Further research on this group of students should aim to determine causal relationships, for instance, by investigating early reading intervention and the potential effect on speech-sound production.

## Introduction

Many children and adults with intellectual disabilities (ID) experience communication difficulties, limiting their participation in daily social activities and education. Having a reduced ability to produce intelligible speech can lead to misunderstandings and frustration and negatively affect both the individual and the communication partner (Drager *et al.* 2010, Light *et al.* 2008). Further, many individuals with ID and communication difficulties do not acquire functional reading skills (Ainsworth *et al.* 2016, Dahlgren Sandberg 2006, Ratz *et al.* 2013). The ability to read and write can provide access to other communication modes and more linguistically advanced augmentative and alternative communication (AAC) systems (Romski *et al.* 2015). Thus, for those with limited functional speech, the ability to read can broaden the opportunity to acquire knowledge and participate in education and social interactions to an even greater degree (Light *et al.* 2011). It is, therefore, important to study both speech production and literacy in the population with ID and communication difficulties, to understand their specific relationship. The specific aim of this research was to investigate the relationship between early literacy skills and speech-sound production in school-aged students with ID and communication difficulties.

Early literacy is defined as the developmental precursor to conventional forms of reading and writing (Castles *et al.* 2018, National Early Literacy Panel 2008). It includes different skills such as phonological awareness (PA) and letter-sound knowledge, which help a child to become a successful reader later in childhood. PA is the ability to reflect on and manipulate the sounds of spoken language, whereas letter-sound knowledge represents the ability to connect orthographical representation to speech sounds. In the present study, early literacy skills refer to PA and letter-sound knowledge. Both PA and letter-sound knowledge have been found to be strongly predictive of and critical precursors for literacy development (Castles *et al.* 2018, Melby-Lervåg *et al.* 2012, National Reading Panel 2000, Scarborough 1998, Tunmer *et al.* 2019). It is well established that PA and letter-sound knowledge skills are lower in groups of children with ID and communication difficulties compared to children with typical development (Ainsworth *et al.* 2016, Fredrick *et al.* 2013). Activities focusing on PA most often require verbal responses, limiting the opportunities for children with ID and communication difficulties to participate in assessments (Barker *et al.* 2014). This means that it may be difficult to disentangle PA from speech difficulties in research. One way to circumvent this challenge is to use PA tests without explicit demands on oral output (e.g. pointing at pictures or using communication devices) (Dahlgren Sandberg 2006, Iacono *et al.* 2004).

In the current study, the aim was to investigate the specific contribution of letter-sound knowledge and PA (early literacy skills) on speech-sound production, after controlling for intellectual level and age, in students of mixed aetiology ID. This was done in a larger sample than in any of the previous studies with a similar aim. There are several theoretical and clinical motivations for the current work. According to Frost *et al.* (2009), functional neuroimaging findings on children aged 6 to 10 years indicated that during the development of reading skills, the brain activates regions that were initially dedicated to processing phonological information in speech to process written language. Psycholinguistic research has revealed that the causal associations between PA, letter knowledge, and speech are likely to be complex – and there are reasons to expect that the acquisition of early literacy skills has implications for aspects of speech, not only vice versa. Specifically, it has been proposed that the knowledge children have about the sound structure of their language creates a bridge between speech and reading processes (Saletta 2015). As a person acquires literacy skills, the awareness of both written and spoken words becomes more apparent (Kolinsky *et al.* 2021). It has been suggested that orthographic representations support speech-sound production by strengthening the precision of the underlying linguistic representations (Brady *et al.* 1994, Huettig *et al.* 2019). Brady *et al.* (1994) incorporated articulatory awareness training with a phonological awareness intervention, and the combination seemed to be a particularly effective way to make the concept of phonemes salient, even for children with reading disabilities. Furthermore, the process of acquiring reading skills in an alphabetic language typically entails sounding out written words, forcing the child to closely monitor phoneme-grapheme correspondence. Interestingly, while phonological representations and speech production skills typically develop prior to literacy development, it is presumed that learning to read and spell fine-tune phonological contrasts can affect the quality of speech (e.g. Gonçalves *et al.* 2020, Rastle *et al.* 2011, Saletta 2015, Saletta *et al.* 2016). Further, PA is included as an important element in speech processing models (e.g. Stackhouse *et al.* 1997), and models of word production often emphasise the importance of precise phonological representations as a basis for creating a phonetic shape (e.g. Dell *et al.* 1999, Levelt 1999). Linking this with the notion that the establishment of orthographic representations improves the precision of and access to phonological representations (Brady *et al.* 1994, Huettig *et al.* 2019), leads to the idea that speech-sound production may be supported by early literacy skills. We may, therefore, expect to see associations between early literacy skills, especially PA, and speech-sound production.

Research that investigates how early literacy skills can predict speech production in students with communication difficulties and/or ID is rather rare but compelling (e.g. Dodd *et al.* 2001, Moriarty *et al.* 2006, Preston *et al.* 2010). Focusing on a non-ID population, Gillon (2000) investigated the efficacy of an integrated PA intervention approach on PA ability, reading performance, and speech production in 61 children with phonological speech difficulties who demonstrated early reading delay. The intervention improved PA ability in the participants, and there was a trend towards improved speech production skills. In Moriarty *et al.* (2006), children with apraxia of speech were included in a PA and letter-sound intervention. Childhood apraxia of speech is characterised by disturbed programming and planning of speech movements, that is, it is a speech condition not (only) reflecting phonological problems but motor aspects. Interestingly the study showed that the intervention not only led to improved word reading skills but also to improved speech accuracy, pointing to the importance of clinicians to ‘concurrently target spoken and written language in children with childhood apraxia of speech’ (p. 732). Dodd *et al.* (2001) emphasise that children with speech impairment are a heterogeneous group, making it unlikely that a single intervention would be appropriate. All in all, while PA is not a prerequisite for good speech for children without speech difficulties, it has been suggested that it may be important for those in need of speech and language therapy and that orthography (letters) may support the process further (Dodd *et al.* 2001, Gillon 2000, Hesketh 2001).

Several studies have revealed associations between aspects of early literacy and speech-sound production in students with ID and communication difficulties (e.g. Barton-Hulsey *et al.* 2018, Burgoyne *et al.* 2021). From practical experience, Light *et al.* (2019) reported improvements in both language and speech production for ten children with mixed aetiology ID and communication difficulties after a reading intervention. Such observations indicate that the orthographic supports improved speech-sound production also in ID (c.f., Brady *et al.* 1994, Huettig *et al.* 2019). The available evidence from formal studies show some inconsistent results, and seem to solely target the Down syndrome population, rather than a wider ID population. Buckley *et al.* (1993) carried out a reading intervention study with 15 participants with Down syndrome and found that both phonology and articulation improved with reading practice. Knight et al. (2015) investigated the impact of reading on the speech-sound production of eight children (aged 11–14 years) with Down syndrome by comparing the pronunciation of words when they were elicited using imitation, picture naming, or reading aloud. When children read the words aloud, they produced significantly more accurate and intelligible speech compared to naming or imitation, ‘providing tentative support for claims that reading words improves accuracy and intelligibility of speech production for children with DS’ (p. 608). Further, Burgoyne *et al.* (2021) found that difficulties with speech-sound production accuracy in a group of 50 children with Down syndrome (aged 5–10 years) were related to age, vocabulary, and reading skills. However, in a follow-up analysis, speech-sound production was observed to be largely stable over 21 months, making it hard to evaluate whether early literacy skills act as a longitudinal predictor of speech-sound development based on the results of that study. Also, a longitudinal study by Laws (2010) on 28 children (aged 5–11 years) with Down syndrome did not reveal that reading skills predicted speech-sound production. Thus, as described above, findings regarding the associations between early literacy skills and speech-sound production are not conclusive for the population with Down syndrome, and we know even less about the wider ID population. The inconsistent results between different studies may possibly be attributed to the small samples used in the previous studies, which varied between 15 and 50. Also, there is certainly great heterogeneity within the samples. In both students with speech-sound difficulties and those with ID, the aetiology of speech difficulties is often complex with multiple linguistic and motor function deficits, which can co-occur and be difficult to separate (Paul 2018). In clinical settings, separating dysarthria from dyspraxia can be difficult and there is comorbidity where the individual can have both diagnoses (Barry 1995, Iuzzini-Seigel *et al.* 2022). For some students, structural (e.g., cleft) and orofacial dysfunction plays an important role for the production of intelligible verbal speech (Mogren *et al.* 2020). Furthermore, age and the overall severity of the intellectual disability can affect speech production (Hammond 1996). Research by Coppens-Hofman *et al.* (2016) showed that ID in childhood was correlated with the degree of speech difficulties in adulthood, regardless of aetiology.

In the current study, a large heterogeneous sample of students with ID and communication difficulties participated; age and intellectual level are, therefore, controlled for in the analyses. Based on previous research and theoretical models of speech and word production (Brady *et al.* 1994, Dell *et al.* 1999, Frost *et al.* 2009, Huettig *et al.* 2019, Levelt 1999, Stackhouse *et al.* 1997), we expect that early literacy skills may explain variance in speech-sound production for the students with mixed aetiology ID and communication difficulties (Buckley *et al.* 1993, Burgoyne *et al.* 2021, Knight et al. 2015, Light *et al*. 2019). A better understanding of associations between early literacy skills and speech may help practitioners and researchers to identify effective interventions for improving both literacy and speech development in school-age children with ID.

The present study will address the following research questions:

‘Is speech sound production associated with PA and letter-sound knowledge in students with ID and communication difficulties?’ In line with the theoretical rationale for the present study and earlier research, we hypothesised that early literacy skills and speech-sound production are positively associated.

‘Do early literacy skills (PA and letter-sound knowledge) explain variability in speech-sound production in students with ID and communication difficulties, after controlling for chronological age and intellectual level?’ Based on the theoretical claims and earlier research on children with Down syndrome (e.g. Buckley *et al.* 1993, Knight et al. 2015), we hypothesised that PA and letter-sound knowledge, both independently and in combination, can explain variability in speech-sound production after controlling for age and intellectual level.

## Method

This cross-sectional quantitative study presents descriptive data, results from correlations, and a regression analysis to answer the research questions. The study is part of Digital Interventions for Literacy Learning, an ongoing project comparing the effects of different digital reading interventions for students with ID in need of AAC. The present study is based on the pre-intervention data from the large project and include about half of the test sessions and test time.

### Participants

A total of 116 students (65 boys and 51 girls) participated. Age ranged from 7 to 21 years with a mean of 13 years and 6 months. All participants attended compulsory schools for students with ID in the western and eastern parts of Sweden and had consented to participate in the Digital Interventions for Literacy Learning project. The teachers were instructed to recruit students (1) with communication difficulties, (2) who were in need of AAC to understand and/or express themselves, and (3) unable to decode words independently and identify a maximum of 20 words. The caregivers who gave their consent responded to a survey on background information about their children. Diagnostic information was collected (see Table 1) from the caregivers. In the Swedish health care system, ICD-10 (World Health Organization 2016) is applied. The student’s level of ID was reported as mild (34%), moderate (53%), or severe (10%) (information was missing for four of the children). Many students had a reported comorbidity with autism, attention deficit hyperactivity disorder, and/or sensory impairment. A large minority (*n* = 28) of the students were also reported to have a rare or unknown diagnosis; caregivers reported a mix of chromosome and genetic disorders and one unknown syndrome. In the group of students with cerebral palsy, five were reported spastic, five dyskinetic, and one without specification. Eleven students were reported to have hearing impairment, ranging from mild to severe. All new-born babies in Sweden are screened for hearing impairment at the hospital and many of them follow medical care program with regular checks. In our study, students in need of hearing aids, used them during testing. Furthermore, 39 students were reported to have a visual impairment, five of whom could not have their vision fully corrected. Separate follow-up analyses were also conducted to examine whether those with reported sensory impairments (hearing or vision) differed from other students in early literacy skills and speech-sound production. Since no difference was seen on any measure, we did not exclude these students or any other students from participation in the study.

**Table 1.** Participants were divided into subgroups according to diagnoses as reported by caregivers, and their used modes of augmentative and alternative communication.

	Only intellectual disability	Autism^a^	Down syndrome^a^	Cerebral palsy^a^	Rare/unknown^a^	Total
Number of students	19	33	25	11	28	116
Nonverbal communication	8	28	17	9	21	83
Manual sign	8	18	24	4	17	71
Pictorial support	12	22	17	8	19	78
Speech-generating device	4	7	4	2	4	21

Note: ^a^Diagnostic categories besides intellectual disability. The category Nonverbal communication includes facial expressions, gestures, eye contact, and body movement. The category Pictures includes pictorial schedules, single pictures, picture communication boards, and pragmatic organized dynamic displays (PODD).

The student’s expressive speech ability varied from limited impairment to significant impairment. All students had access to some sort of AAC in their daily school environment, either for expressive support, comprehension support, or both (Table 1). The use of AAC varied widely, from nonverbal communication, manual signs, pictorial support, to technology-based tools such as speech-generating devices. Most students also used more than one AAC mode. For more detailed information about diagnoses and communication modes, see Table 1.

### Procedure and materials

The students were tested individually at their schools, in a silent environment. The students had the opportunity to have a teacher or assistant present during the assessment. The first, third, and fourth authors administered tests. Two were experienced speech and language pathologist, and one had experience from behavioural and psychological testing as a researcher. Test administrators practiced the test procedure before data collection began. The test order was visualised and presented to the student using a pictorial schedule. All students were tested with the same material. The test session lasted about 45–75 min, including short breaks. During breaks, students were given the option to choose activity, for example, having a small snack, water, or the option to move around in the room for a few minutes. To help them decide, they were shown both pictures and samples of the snack options to choose from.

Tests on PA and letter-sound knowledge were modified to enable participation for individuals in need of AAC, eliminating speech production as the only response option. A more detailed description is presented under the specific tests. Picture communication and manual signs were used in combination with verbal instructions as needed during the test session.

#### Speech-sound production

Speech-sound production was tested with a subtest from the Assessment of Phonology, an instrument for the production of phonemes (Frylmark 2015). The Assessment of Phonology contains several modules to assess speech-sound production, five action pictures, and a single picture booklet. For this study, an action picture of a café visit was chosen to elicit oral speech. This picture has 28 target words, representing 138 phonemes, including all Swedish phonemes, but not in every position of the word. All students had reached the age of six, at which time all Swedish children are typically expected to be able to produce all Swedish phonemes (Blumenthal *et al.* 2014).

The student was first asked to spontaneously describe the action picture orally. The goal was to collect all 28 words with the students’ spontaneous speech. If this was not possible, the administrator pointed at the specific objects or actions that were not spontaneously named. Prompting was used as a last option, and the student was then asked to repeat the label of the object or action, as pronounced by the administrator. Test sessions were video recorded, and a phonemic transcription of all target words was performed afterwards by first and fourth author. Only oral responses were analysed. No output from speech-generating devices was included in this assessment.

#### Phonological awareness

For the assessment of PA, three subtests from MiniDUVAN (Wolff 2013) were chosen: Rhyme identification, Phoneme identification, and Phoneme synthesis. Each subtest had two practice-items with feedback, followed by nine test items. To overcome speech intelligibility difficulties, all subtests had a nonverbal response with pictures. A subtest was terminated if the student gave an incorrect response to three consecutive items.

In Rhyme identification, the student answered yes/no when asked whether two words rhymed (e.g. ‘Does hat – cat rhyme?’) using the student’s preferred mode of answering. The student could either point to pictures representing yes (thumb up) and no (thumb down), use a personal AAC mode, or choose to use spoken responses. One point was awarded for each correct response for a maximum of nine points.

In Phoneme identification, the student was presented with a picture of the sun and asked to point to another picture with an object with the same initial phoneme in its name (e.g. ‘This is a sun. Sun starts with/s/. Point to the picture that start with the same sound/s/’.). For each item, the child could choose from three pictures when responding. The target phoneme was the same for all items. For each correct selection, one point was awarded for a maximum of nine points.

In the last subtest, Phoneme synthesis, the administrator pronounced three to five sounds for the student who was asked to assemble the sounds into one word. In the standardised test procedure, only verbal responses are allowed. However, due to the speech difficulties of the participants in the present study, an adapted response procedure was applied with pictures. For each item, three pictures, one representing the correct word, were presented to the student and the student responded by pointing to one of the pictures. For each correct response, one point was awarded, maximum of nine points. The dependent variable for PA was the total number of correct responses across the three subtests (0–27).

#### Letter-sound knowledge

The letter-sound knowledge task was constructed for use in the present study. The task contained eight target letters, half of which were consonants and half vowels. Four were presented as capital letters (*L, D, A*, and *Ö*) and four as lowercase letters (*g, o, k*, and *å*), and were selected based on production place, manners, and graphic representation. The task included eight items. In each item four letters were presented in Arial font, size 130, on a sheet of paper. The target letters *L* and *D* were presented together with *E* and *T*, and *A* and *Ö* were presented together with *H* and *B*. Lowercase *g* and *o* were presented together with *i* and *r*, and *k* and *å* together with *u* and *p*. At presentation, the student was asked to point to the letter corresponding to a sound (e.g. /*l*/,/*d*/) uttered by the test administrator. The dependent variable was the total number of sounds correctly paired to the corresponding letter (0–8).

#### IQ

As a proxy of the participants’ general IQ, Raven’s 2 was used (Raven 2019). This test is suitable for nonverbal children with communication-related difficulties and offers a set of items applicable to the lower end of intellectual ability. Students were asked to point, aided or unaided, to one of the five pictures that completed the presented figure in the paper version. After six successive failures, the task was ended. Modules A, B, and C were administered, including 36 items, each being awarded one point if correctly solved. Based on the total number of correct items, IQ (around a mean of 100 and a standard deviation of 15) was estimated according to age norms reported in the manual.

### Speech analysis and reliability

Transcriptions were performed by either the first or fourth author, which was the same author that administered the test for the respective student. All target words could be listened to repeatedly. Each phoneme in all 28 words was scored as either correctly produced or incorrectly produced. Both place and manner of articulation had to be correct. All substitutions and omissions were scored as incorrect. If a child added morphological endings to the target word, those were not analysed (e.g. if a student said ‘shoes’ for shoe, the plural was not scored). For each correctly produced phoneme, one point was awarded. In line with Shriberg *et al.* (1997), the dependent variable, percentage phonemes correct, was calculated by dividing the number of correctly produced phonemes by the total amount of possible correct phonemes and then multiplying by 100 (0–100). The total amount of points (max 138) was used for calculating the final score for percentage phonemes correct.

An interrater reliability analysis on speech-sound production (percentage phonemes correct) was calculated on a subset (*n* = 20) recording. To determine the consistency between the two raters, ten students from each of the two transcribers were randomly selected. The subset represented all five diagnostic subgroups (ID = 2, autism = 6, Down syndrome = 7, cerebral palsy = 2, rare/unknown = 3).

Interrater reliability for percentage phonemes correct across both transcribers was first calculated with the intra-class correlation coefficient. The average intra-class correlation coefficient was 0.997 with a two-way mixed method and absolute agreement. Second, a point-by-point interrater agreement on all 138 phonemes between both transcribers was also calculated with Cohens Kappa. There was substantial agreement between the two raters, (*κ* = 0.78) (Landis *et al.* 1977). To meet the requirement for reliability regarding the tests for PA and letter-sound knowledge that did not follow standard performance, the test-retest reliability was calculated. A group of 25 students was retested after six weeks and after twelve weeks. Test-retest scores indicated high reliability for both PA (six weeks, *r* = 0.89; twelve weeks, *r* = 0.92) and letter-sound knowledge (six weeks, *r* = 0.90; twelve weeks, *r* = 0.85).

### Statistical analysis

The data was processed with descriptive, correlation, and multiple regression analysis. Parametric (Pearson *r*) and nonparametric (Spearman *r_s_*) correlations between the variables were estimated and compared. The results were similar regardless of method, and a parametric approach was, therefore, applied. To control for possible influences of different diagnoses and sensory impairments, we performed group comparisons with Mann–Whitney *U* Tests, adjusted for multiple group comparisons.

To investigate the associated variance of early literacy skills on speech-sound production (percentage phonemes correct), a multiple regression analysis was performed. To remove any effects related to general developmental differences, age and IQ were added in the first block using the enter method. In a second block, PA and letter-sound knowledge were added by using the enter model building strategy. The alpha-level was set to 0.05 in all analyses, and no correction was applied for multiple statistical tests. Collinearity statistics for the multiple regression did not indicate multicollinearity. A Durbin − Watson test (2.088) indicated that the residuals were independent (Field 2013). The following guidelines were used for interpretation of *R*^2^: very weak = < 0.02, weak = 0.02 − 0.13, moderate = 0.13 − 0.26, and substantial = > 0.26 (Cohen 1988). Statistical analyses were calculated in IBM SPSS Statistics 28.

### Ethical considerations

The present study was approved by the Swedish Ethical Review Authority (case number 2019-03845 and 2020-06215). Students and caregivers received written information supported with pictures and the caregivers gave their written consent for the students to participate in this study. Data was pseudonymised and the World Medical Association Declaration of Helsinki was followed. The students’ verbal and nonverbal communication was always respected, and testing was interrupted at any sign of discomfort.

## Results

All 116 students completed the full test battery of speech-sound production, PA, letter-sound knowledge, and IQ. Table 2 presents a summary of means, standard deviations, 95% confidence intervals, and correlations between the measures. There was a widespread ability in speech-sound production. As a group, the students produced 66.5% of the phonemes correct but with a wide range from 0 to 100%.

**Table 2.** Descriptive statistics and correlations between participants’ (*n* = 116) speech production – percentage phoneme correct, IQ, age, phonological awareness, and letter-sound knowledge.

Variables	*M*	*SD*	95% CI		1	2	3	4	5
			*LL*	*UL*					
1. Percentage phoneme correct	66.5%	32.4%	60.5%	72.4%	–	.14	.13	.54**	.45**
2. IQ	48.8	13.3	46.4	51.3		–	–0.23*	.20*	.23*
3. Age	13.6	3.2	13.0	14.2			–	.29**	.24*
4. Phonological awareness	12.7	8.0	11.3	14.2				–	.60**
5. Letter-sound knowledge	6.3	2.5	5.9	6.8					–

Note. CI: confidence interval; LL: lower limit; UL: upper limit.

* *p* < .05.

** *p* < .01.

### Relationships between speech, PA, letter-sound knowledge, age, and IQ

Pearson correlation analyses were conducted to examine the relationship between speech-sound production, IQ, age, PA, and letter-sound knowledge. Correlations indicated that speech-sound production (percentage phonemes correct) was positively correlated with PA (*r* = 0.54 *p* < .001) and letter-sound knowledge (*r* = 0.45, *p* < .001). Moreover, there was a significant correlation between PA and letter-sound knowledge (*r* = 0.60 *p* < .001). Conversely, speech-sound production did not indicate significant associations with chronological age and IQ.

### Speech, PA, and letter-sound knowledge

To answer the question of whether PA and letter-sound knowledge were associated with speech-sound production in the multivariate analyses, a hierarchical multiple regression model was conducted. Table 3 presents the multiple regression model in two steps. In Step 1, age and IQ were entered to control for their effects, and the results indicated that these variables explained a very small amount of variance in percentage phonemes correct, *F*(2,113) = 2.875, *p* = .061, *R^2^*_adj_ *_=_* 0.03. The specific contributions of age and IQ on speech-sound production were marginal.

**Table 3.** Hierarchical multiple regression model with speech production PPC as dependent variable.

Variable	*B*	95% CI for *B*		*SE B*	*β*	*R* ^2^_adj_	Δ*R*^2^_adj_	*t*	*p*
		*LB*	*UB*						
Block 1						0.03	0.03		
Constant	21.16	–16.61	58.93	19.07				1.11	.269
Age	0.005	0.00	0.01	0.003	0.17			1.85	.067
IQ	0.44	–0.01	0.90	0.23	0.18			1.94	.055
Block 2						0.29	0.26		
Constant	30.73	–2.24	63.70	16.64				1.85	.067
Age	–0.001	–0.01	0.004	0.002	–0.04			–0.44	.662
IQ	0.01	–0.40	0.42	0.21	0.003			–0.04	.967
PA	1.77	0.94	2.59	0.42	0.43			4.25	<.001
LSK	2.72	0.02	5.42	1.36	0.20			2.00	.048

Note. CI: confidence interval; LB: lower bound; UB: upper bound; IQ: intelligence quotient, PA: phonological awareness; LSK: letter-sound knowledge.

In Step 2, PA and letter-sound knowledge were added, and the results showed that together they accounted for 29% of the variance in the phoneme production, *F*(4,111) = 12.791, *p* < .001, *R^2^*_adj_ = 0.29. Looking at the specific individual contributions of the early literacy skills predictors, the results showed that PA and letter-sound knowledge are uniquely and positively associated with speech-sound production. This indicates that higher scores on PA and letter-sound knowledge are associated with higher percentage phonemes correct score.

The present study was designed specifically to focus the relationship between pre-literacy skills and speech-sound production. However, to explore the potential effects of confounds related to the heterogeneity of the sample, subsequent analyses were performed to control that the performance on the outcome measure (percentage phonemes correct) and the predictors of special interest (PA, letter-sound knowledge) did not differ depending on sensory impairment (hearing impairment and visual impairment) and/or diagnosis. Nonparametric group comparisons, adjusted for multiple tests, revealed that no subgroup (diagnosis or sensory impairment) performed worse or better than the rest of the sample. Thus, the heterogeneity of the sample does not likely explain the results in the main analysis.

## Discussion

The present study aimed at determining the specific contribution of letter-sound knowledge and PA (early literacy skills) to speech-sound production, after controlling for intellectual level and age, in students of mixed aetiology ID. The main finding was that early literacy skills (PA and letter-sound knowledge) explain 29% of the variance in speech-sound production, after controlling for age and IQ, in a heterogeneous sample of children with ID and communication difficulties.

### Correlations between speech-sound production and early literacy skills

The first question of this study was whether there is a relationship between speech-sound production and early literacy skills. The correlations indicated that speech-sound production was positively correlated with both PA and letter-sound knowledge, which indicates that there is a connection between these variables in students with mixed aetiology ID. This may suggest that better speech-sound production and good PA abilities are both indicative of strong phonological representation. Similar results were found by Preston *et al.* (2010), who investigated pre-schoolers with speech-sound disorders and typical cognitive development. Thus, our results may reflect a general association not specific to students with ID. Preston *et al.* results also indicated that participants who produced more atypical speech-sound errors performed more poorly on PA tasks. In addition, we saw that speech-sound production was positively associated with letter-sound knowledge, a result inconsistent with the findings of Barton-Hulsey *et al.* (2018). They did, however, find a small to moderate, but nonsignificant correlation (*r* = 0.299) between speech production and letter-sound knowledge in pre-school aged children with ID. Besides differences in age between the sample in Barton-Hulsey and in ours, another, perhaps more important, difference concerned the sample size (*N*** **=** **42 and *N*** **=** **116, respectively). Thus, we were more likely to detect smaller associations, but also to get better statistical estimates. Another important difference was that Barton-Hulsey *et al.* (2018) measured oral motor skills, motor speech-sound production, and speech intelligibility with the Kaufmann Speech Praxis Test for Children, while, in the present study, speech-sound production was measured in percentage phonemes correct in 28 single words. The assessment procedures may have tapped into different facets of the children’s speech production ability, which may explain the different results between studies.

The present study included a diverse range of medical conditions that are associated with motor disabilities, such as cerebral palsy. Previous research has shown a relationship between speech-sound production, phonological awareness, and letter-sound knowledge in children with cerebral palsy and communication difficulties (Peeters *et al.* 2009, Vandervelden *et al.* 1999). The results of our small group of students with cerebral palsy (*n* = 11) was comparable to the rest of the students in the study on speech-sound production, phonological awareness, and letter-sound knowledge. This suggests that the decision not to exclude this subgroup from the main analysis is unlikely to have confounded the results.

A study by Burgoyne *et al.* (2021), which included a sample of children with Down syndrome, reported results that were more similar to ours in terms of correlation patterns. More specifically, they found a positive correlation between speech-sound production and letter-sound knowledge. Also, Burgoyne *et al.* found that a phoneme blending variable, tapping into PA, remained significantly correlated with speech-sound production after controlling for age. Moreover, they did not find any correlation between speech-sound production and IQ in their group of participants, which is in line with our findings. However, in contrast to our study, they did find a positive correlation between speech-sound production and age. The participants in Burgoyne *et al.*’s study were younger (5–10 years of age) than the participants in our sample and at a developmental stage where speech is generally expected to develop further, also in a group with ID. Further, in a study by Mogren *et al.* (2020), which included a group of children (6–16 years of age) with speech sound disorder but without ID, a significant correlation was found between consonant production and age, but age alone did not explain the variability in consonant production. Collectively, these findings suggest that children with ID and communication difficulties can continue to develop their speech-sound production after entering school, but not at the same speed as when they were younger.

There was a strong statistically significant correlation between PA and letter-sound knowledge in the present study. This correlation was expected since it is an established finding in the literature on early literacy skills (Tunmer *et al.* 2019) and it was earlier shown by Barton-Hulsey *et al.* (2018), in a group of pre-school children with ID.

### The prediction of early literacy skills on speech-sound production

As described above, we found evidence of positive associations between speech-sound production and early literacy skills. The second question of the present study was to determine if early literacy skills accounted for variability in speech-sound production beyond the influence of IQ and age. The results indicated that a substantial proportion (Cohen 1988) of the variance in speech-sound production could be accounted for by early literacy skills (PA and letter-sound knowledge) in the present study. This suggests that students with ID of mixed aetiology and with better early literacy skills, specifically PA and letter-sound knowledge, are also more likely to have better speech-sound production skills. This finding lends support to theoretical models of speech and word production (Brady *et al.* 1994, Dell *et al.* 1999, Frost *et al.* 2009, Huettig *et al.* 2019, Levelt 1999, Stackhouse *et al.* 1997), as applied in this case to students with ID, and corroborates previous research conducted on children with Down syndrome by Buckley *et al.* (1993), Burgoyne *et al.* (2021), and Knight et al. (2015). Furthermore, our results are in line with the clinical experiences of speech-language pathologists and teachers working with children with ID and communication difficulties. For example, Light *et al*. (2019) described how children with ID and complex communication needs improved their speech-sound production precision as a possible effect from participating in a reading intervention. However, this has only been partly confirmed in studies on literacy interventions in this group. Individuals with ID and communication difficulties represent a heterogeneous group, even when the genetic causes are the same, making it difficult to draw general conclusions for the entire group of ID, or even within a Down syndrome-specific group. Still, the results from this study indicate that early literacy skills instruction is important to include in the education of students with ID and communication difficulties in need of AAC, not only for literacy learning, but potentially also for the development of speech. In terms of clinical and educational implications, the current study is potentially important by revealing associations between pre-literacy skills and quality of speech output in students with ID. More research is needed as regards the causal influences. Stating this important main conclusion from our study, it is of equal importance to say that our study does not give the whole picture. Not all children will be able to develop their speech, despite developing early literacy skills, which has been found in studies of children with cerebral palsy and significant motor-speech impairments (Dahlgren Sandberg 2006, Moriarty *et al.* 2006). Thus, although our study does not give the whole picture, we think it provides an important piece of knowledge to this area.

### Strengths and limitations

Students in this study represented a heterogeneous yet representative sample of the target population: students with ID of mixed aetiology and communication difficulties enrolled in Swedish compulsory schools for students with ID. The study’s greatest strength was the inclusion of a large representative sample (*N*** ***=*** ***116*) of students in this population. This population otherwise tends to be studied in small samples or excluded from research altogether due to problems in recruiting participants and testing them on relevant measures. Nevertheless, we did explore whether heterogeneity such as sensory impairments and autism seemed to influence the results, and found that it likely had little, if any, effect on the general pattern of results.

There are a few of tests that can be used when participants do not have access to verbal speech. For the population included in the present study, materials with other response options were crucial to achieve a fair test of ability level. Students were, therefore, tested with materials, adapted for students in need of AAC. As described in the methods section, verbal problems were dealt with by providing pictorial support, as well as by providing adaptations to the specific student’s communicative, motoric, and cognitive needs during the test situation (e.g. to maintain motivation and concentration). When choosing the tests, degree of difficulty, length of performance, and the ability to answer without speech were considered. The procedure we used to assess early literacy skills without the use of speech is essential for students with ID and in particular for those with additional speech disorders. In addition, the absence of demands for speech output in the PA and letter knowledge assessments mean that the observed associations with speech output are not a mere assessment artefact.

However, even with the adaptations, the test battery had some limitations. First, to avoid fatigue, the number of tests that we used was kept at a minimum. This meant that not all phonemes and positions of articulation could be tested, and that specific tests of language comprehension and of oral-motor functions were not included. Second, the test used to assess letter-sound knowledge included only eight of the 28 Swedish letters, and therefore, also covered a limited set of articulation and manners. Future studies on the associations between speech-sound production and early literacy skills should take these limitations into consideration.

## Conclusion and future directions

This study adds to the small amount of research regarding speech-sound production and early literacy skills in students with ID, and specifically contributes to our understanding of the associations between speech-sound production and early literacy skills in students with ID with mixed aetiology. We tentatively conclude that the development of literacy may play a role in developing speech-sound production skills in students with mixed aetiology ID, similar to what some studies have reported för children with Down syndrome and children with ID and mixed aetiology (Buckley *et al.* 1993, Knight et al. 2015, Light *et al*. 2019).

Given the observed associations in the present study, further investigations are warranted to determine whether literacy intervention can produce positive effects on speech-sound production in children with ID. In future studies, investigating the relationships between speech-sound production and early literacy skills, comparisons should be made for groups of different ID aetiology, with and without additional diagnoses such as autism, speech motor function, and across different age bands.
